# BARRIERS AND FACILITATORS TO IMPLEMENTING MEDICARE’S CHRONIC CARE MANAGEMENT IN RURAL PRIMARY CARE

**DOI:** 10.1093/geroni/igad104.2962

**Published:** 2023-12-21

**Authors:** Elizabeth Punke, Barbara Dabrowski, Abby Teply, Katy Richardson, Christine McKibbin, Catherine Carrico

**Affiliations:** University of Wyoming, Laramie, Wyoming, United States; University of Wyoming, Laramie, Wyoming, United States; University of Wyoming, Laramie, Wyoming, United States; University of Wyoming, Laramie, Wyoming, United States; University of Wyoming, Laramie, Wyoming, United States; University of Wyoming, Laramie, Wyoming, United States

## Abstract

In 2015, the Centers for Medicare and Medicaid Services introduced Chronic Care Management (CCM) to provide reimbursement to practices for care coordination services necessary to manage chronic conditions among older adults. However, uptake of this program among primary care practices is low, and few Medicare beneficiaries actually receive this service. Implementation of CCM may be even more challenging in rural, low-resourced areas. The purpose of this study was to examine factors associated with more and less successful implementation of CCM within primary care practices located in a rural state. A qualitative study was conducted, which was guided by the Consolidated Framework for Implementation Research (CFIR). Employees of practices (n = 8) that have implemented CCM were approached for study participation. Members (n = 17) of various healthcare teams were included in the study. Participants completed sociodemographic, professional, and practice history measures and completed a qualitative interview. Participants (n = 17) were predominantly White (n = 16; 94%), female (n = 16; 94%), and represented care coordinators, primary care providers, allied health professionals, and administrators. CrossTX records were analyzed to categorize practices by high and low implementation success. Content analysis, guided by CFIR, identified domains associated with implementation success, including Intervention Characteristics (I.e., Relative Advantage), Outer Setting (e.g., Patient Needs and Resources), Inner Setting (e.g., Implementation Climate, Leadership Engagement), and Process (e.g., Champions). Strategies to address barriers include addressing characteristics of the Inner Setting (e.g., Available Resources). Developing training mechanisms to address staff turnover is critical to address barriers.

